# Supplementary material for: Combined Newborn Screening Allows Comprehensive Identification also of Attenuated Phenotypes for Methylmalonic Acidurias and Homocystinuria
Source: Nutrients. 2023 Jul 28;15(15):3355. doi: 10.3390/nu15153355 (PMC10420807; doi:10.3390/nu15153355)
Supplement: Supplementary file 1 [file nutrients-15-03355-s001.zip › nutrients-2458719-supplementary.pdf]

Supplementary Table S1.

In 42 cases, not the suspected diagnosis from the NBS sample but another diagnosis of the combined algorithm was confirmed first by the biochemical diagnostics and second, for IMDs only, by genetic testing.

N	Suspected diagnosis in NBS	Confirmed diagnosis	Mode of confirmatory diagnostics
**1**	Propionic acidemia	Neonatal Vitamin B_{12}-Deficiency	Biochemical
**2**	Propionic acidemia	Methylmalonic aciduria	Biochemical and genetical
**3-7**	Methylmalonic aciduria	Neonatal Vitamin B_{12}-Deficiency	Biochemical
**8-33**	Isolated Remethylation Disorders	Neonatal Vitamin B_{12}-Deficiency	Biochemical
**34-35**	Combined Remethylation Disorders	Neonatal Vitamin B_{12}-Deficiency	Biochemical
**36-41**	Homocystinuria	Neonatal Vitamin B_{12}-Deficiency	Biochemical
**42**	Neonatal Vitamin B_{12}-Deficiency	Propionic acidemia	Biochemical and genetical
